# A model to estimate insulin sensitivity in dairy cows

**DOI:** 10.1186/1751-0147-49-29

**Published:** 2007-10-11

**Authors:** Paul Holtenius, Kjell Holtenius

**Affiliations:** 1 Department of Clinical Sciences, Swedish University of Agricultural Science (SLU), SE-75007, Uppsala, Sweden; 2 Department of Animal Nutrition and Management, Kungsängen Research Center, Swedish University of Agricultural Science (SLU), SE-753 23 Uppsala, Sweden

## Abstract

Impairment of the insulin regulation of energy metabolism is considered to be an etiologic key component for metabolic disturbances. Methods for studies of insulin sensitivity thus are highly topical. There are clear indications that reduced insulin sensitivity contributes to the metabolic disturbances that occurs especially among obese lactating cows. Direct measurements of insulin sensitivity are laborious and not suitable for epidemiological studies. We have therefore adopted an indirect method originally developed for humans to estimate insulin sensitivity in dairy cows. The method, "Revised Quantitative Insulin Sensitivity Check Index" (RQUICKI) is based on plasma concentrations of glucose, insulin and free fatty acids (FFA) and it generates good and linear correlations with different estimates of insulin sensitivity in human populations. We hypothesized that the RQUICKI method could be used as an index of insulin function in lactating dairy cows. We calculated RQUICKI in 237 apparently healthy dairy cows from 20 commercial herds. All cows included were in their first 15 weeks of lactation. RQUICKI was not affected by the homeorhetic adaptations in energy metabolism that occurred during the first 15 weeks of lactation. In a cohort of 24 experimental cows fed in order to obtain different body condition at parturition RQUICKI was lower in early lactation in cows with a high body condition score suggesting disturbed insulin function in obese cows. The results indicate that RQUICKI might be used to identify lactating cows with disturbed insulin function.

## Findings

In humans the prevalence of obesity, overweight and other food related problems are increasing in many areas in the world with a number of metabolic diseases as a consequence [1]. There is general agreement that the etiologic key component in the pathogenesis of these metabolic diseases is insulin resistance (IR). Insulin resistance is defined as a condition when higher than normal insulin concentrations are needed to achieve normal metabolic responses [2]. One general effect of disturbed insulin function in man is infiltration of fat in the liver which in turn may give rise to a number of pathological changes [3,4]. Also in dairy cows obesity and fatty liver occur frequently and the fat cow syndrome is a well-known problem [5].

The importance of IR as the primary etiological factor in the development of metabolic disturbances has increased the interest for measurement of insulin sensitivity. It is not possible to estimate insulin sensitivity only by determination of the plasma concentration. Different kinds of glucose tolerance tests (GTT) used in clinical investigations need time and are not suitable for epidemiological investigations. In lactating dairy cows about 80 % of the cellular glucose uptake occurs independently of insulin [6], which reduces the suitability of GTT. Furthermore, due to the digestive physiology of adult ruminants it is generally difficult to prepare steady state protocols.

A method for epidemiological studies of insulin sensitivity in man has been developed [7,8]. The method, "Revised Quantitative Insulin Sensitivity Check Index" (RQUICKI) implies a valuation of the homeostatic energy balance and is based on plasma concentrations of glucose, insulin and free fatty acids (FFA). RQUICKI generates good and linear correlations with direct euglycemic hyperinsulinemic measurement of insulin sensitivity as well as with other estimates of insulin sensitivity in different human populations [8]. RQUICKI is calculated based on the blood plasma concentrations of glucose (Gb) in mg/dl, insulin (Ib) in μU/ml and free fatty acids (FFAb) in mmol/l, b denotes basal values. The formula was first described by Perseghin et al. [7] in the following manner: RQUICKI = 1/[log (Gb) + log (Ib) + log (FFAb)]. A low index value indicates decreased insulin sensitivity.

We hypothesize that RQUICKI could be used as an index of insulin function in dairy cows. The first aim of the present study was to monitor normal variations in RQUICKI during the period from calving to mid-lactation in high producing dairy cows without clinical signs of disease. Secondly the aim was to investigate how the RQUICKI post partum was affected in cows subjected to different dry-period feeding regimen thus creating different body condition at parturition.

We have elaborated data from a previous investigation [9]. Twenty herds and 237 dairy cows were examined. Each herd was visited once during the housing season and all cows within the first 15 weeks of lactation were included in the study. Blood plasma was analysed for glucose, FFA and insulin as previously described [9]. RQUICKI was calculated as described by Perseghin et al. [7]. Before statistical analysis, the data were divided into the five lactation periods i.e. period 1, samples taken from parturition until the 3rd lactation week, period 2; lactation weeks 4–6, period 3; lactation weeks 7–9, period 4; lactation weeks 10–12 and period 5; lactation weeks 13–15. The plasma components and RQUICKI were statistically analysed using the general linear models procedure (Minitab release 14). The model accounted for herd and lactation period.

In the second study we elaborated data from another previously performed experiment [10,11]. In that study the influence of body condition at parturition on metabolism and performance during lactation was investigated. Body condition was evaluated as described by Agenäs et al. [10] and FFA, insulin and glucose were analysed as described by Holtenius et al. [11]. RQUICKI was calculated as described above.

**Figure 1:**
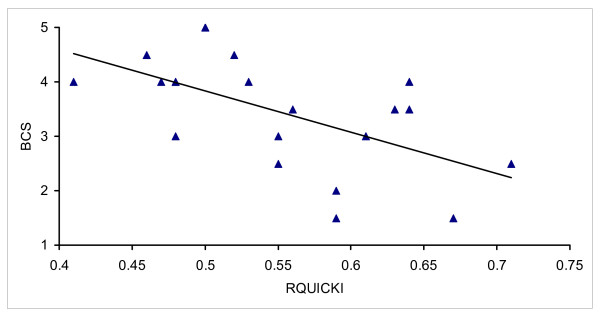
Relationship between body condition score (BCS) and RQUICKI. BCS was determined on a five-grade scale with half point increments where 1 represents very thin and 5 an obese animal.

The plasma concentrations of glucose, insulin and FFA and the index RQUICKI during the 15 weeks after parturition are presented in Table 1. Glucose, insulin and FFA were all significantly affected by sampling week relative to parturition. The glucose level was lowest in samples taken during the first three weeks after calving thereafter only small fluctuations occurred. The concentration of insulin was also lowest during the period including the first 3 weeks after parturition and the highest level was observed 10 – 15 weeks after parturition. The plasma concentration of FFA was highest within three weeks after calving and decreased gradually thereafter. These changes agree with the results of other studies [11-13]. The influence of lactation week on these plasma components makes them less suitable as individual indices to identify changes in insulin sensitivity. On the other hand the RQUICKI was not significantly affected by lactation week. The result thus suggests that RQUICKI in contrast to the individual components insulin, FFA and glucose, was not affected by the homeorhetic adaptations in energy metabolism that occurred in apparently healthy cows during lactation. This would facilitate the use of RQUICKI as an index to identify changes in insulin sensitivity. In humans obesity is related to a reduced insulin sensitivity concomitant with an increased incidence of metabolic diseases [8,1]. We have previously observed that cows that were overfed during the dry period, and thus having a high body condition score (BCS) showed a reduced glucose disappearance after a GTT and it was suggested that the cows with a high body condition score were more insulin resistant than the thinner cows [11]. Interestingly, using the same data set we here found that there was a significant negative linear relationship between body condition score and RQUICKI (P = 0.003) (Figure 1). The results are thus in agreement with the findings in humans [8].

**Table 1:** Glucose, insulin and FFA in plasma and RQUICKI -values in cows during the first 15 weeks of lactation. N = 237 cows. Data presented as means ± standard error of the mean.

	Lactation week					
Item	1–3	4–6	7–9	10–12	13–15	P-value

Glucose (mmol/l)	3.05 ± 0.10	3.21 ± 0.10	3.30 ± 0.10	3.39 ± 0.11	3.20 ± 0.08	0.03
Insulin (μU/ml)	6.7 ± 0.9	8.6 ± 0.9	8.4 ± 0.9	10.4 ± 0.8	10.1 ± 1.1	0.006
FFA (mmol/l)	0.41 ± 0.03	0.29 ± 0.03	0.22 ± 0.03	0.20 ± 0.03	0.17 ± 0.03	<0.001
RQUICKI	0.48 ± 0.15	0.50 ± 0.15	0.51 ± 0.15	0.50 ± 0.12	0.52 ± 0.17	0.28

## Conclusion

We have demonstrated that RQUICKI was not affected by the adaptations that normally occur in apparently healthy cows during the first months of lactation. However, RQUICKI was lower, reflecting disturbed insulin function, in obese cows. The results indicate that RQUICKI might be used to identify cows with disturbed insulin function. However evidence of a relationship between RQUICKI and metabolic diseases in dairy cows is yet lacking.

## Competing interests

The author(s) declare that they have no competing interests.

## Authors' contributions

PH came up with the idea. The manuscript was drafted in co-operation. Both authors approved the final manuscript
